# Seed germination of *Bidens subalternans* DC. exposed to different environmental factors

**DOI:** 10.1371/journal.pone.0233228

**Published:** 2020-05-14

**Authors:** Juliana de Paiva Pamplona, Matheus de Freitas Souza, Danielle Marie Macedo Sousa, Hélida Campos de Mesquita, Claudia Daianny Melo Freitas, Hamurábi Anizio Lins, Salvador Barros Torres, Daniel Valadão Silva

**Affiliations:** 1 Department of Agronomic and Forest Sciences, Universidade Federal Rural do Semi-Árido, Mossoró, Rio Grande do Norte, Brazil; 2 Department of Agronomic, Instituto Federal de Educação, Ciência e Tecnologia do Rio Grande do Norte, Apodi, Rio Grande do Norte, Brazil; Chinese Academy of Forestry, CHINA

## Abstract

*Bidens subalternans* DC. is a weed found in several tropical countries such as Brazil. Large number of produced seeds and easy dispersion favor the colonization of agricultural fields by this species. To know the factors that affect the germination of *B*. *subalternans* can help to understand its ecology, permitting to develop control strategies. Laboratory experiments were carried out to evaluate how the temperature, photoperiod, burial depth, water deficit, and salt stress affect the seed germination of *B*. *subalternans*. The means of the treatments of each experiment were shown in scatter plots with the bars indicating the least significant difference (LSD, p≤0.05). The results showed a germination percentage above 77% for a wide alternating temperature (15/20 C to 30/35 C night/day). The highest germination and uniformity occurred at 25/30°C night/day. Only 11% of the seeds germinated at a temperature of 35/40°C night/day. The deeper burial of seeds reduced their germination. Only 17% of the seeds germinated in darkness conditions. However, in constant light and 12 hours of light/dark conditions the germination percentage was over 96%, confirming the light dependence of the *B*. *subalternans* during germination. In constant light and 12 hours of light/dark, the germination was over 96%. *B*. *subalternans* seeds showed sensitivity to water and salt stress, and their germination was inhibited under a water potential of -0.4 MPa and 100.09 mM, respectively. The sensitivity of *B*. *subalternans* seeds to high temperatures, water stress, and salt stress explains the high frequency of this weed in south-central Brazil. The light and sowing depth showed that burial of seeds by mechanical control is a strategy to reduce the high infestation of *B*. *subalternans*.

## Introduction

*Bidens subalternans* DC. (Beggarticks) is a weed native of South America and infests annual and perennial crops. This species has high similarity to species *Bidens pilosa* L., also belong to the genus *Bidens* with a widespread incidence in agricultural fields [1, 2, 3]. The incidence of *B*. *subalternans* in Brazilian agricultural areas increased from 1996 when biotypes resistant to acetolactate synthase (ALS) inhibiting herbicides group were identified in South America [4]. Currently, this species is observed in all Brazilian regions, even in semi-arid, which suggests the excellent adaptation of this species to different environmental conditions [5].

The high seed production (up to 6000 per plant) and easy dispersion due to the presence of bristles permit *B*. *subalternans* to colonize cultivated and uncultivated areas [6]. Also, the seeds dormancy ensures the infestation during several crop cycles. After seed germination, *B*. *subalternans* have a high capacity to extracted water and nutrients from the soil, making this species a good competitor by growth resources, reducing crop yield [7]. [8]. reported a reduction of 30% in the soybean yield caused by weed interference. According to the same authors, this yield loss occurred due to the high density of *B*. *pilosa* and *B*. *subalternans* in the fields.

The chemical method has been adopted to control *B*. *subalternans* population, similar to the strategies used for *B*. *pilosa*. However, sequential applications of ALS-inhibitors and photosystem II herbicides have increased the frequency of biotypes with multiple resistance [9, 10]. The solution to this problem is the integrated management, involving others control methods for *B*. *subalternans*. The first step to select efficient control methods is to study the ecology of the species, especially aspects related to their establishment in crop fields. Among these aspects, the germination and emergence, as well the factors that affect them, should be investigated because they can provide valuable information, permitting to develop management strategies [11].

The germination and establishment of weeds in agricultural fields are affected by several environmental factors. Among these factors, water availability, salinity, and temperature may be determinant for weeds to succeed in adapting to new sites [12, 13]. For *B*. *subalternans*, the effect of these factors on germination process until was not clearly investigated. In addition to these factors, luminosity and burial depth also may alter seed germination. For example, some species present higher germination when their seeds stay on the soil surface. In contrast, others can germinate even in deeper regions of the ground, such as *Stellaria media* (L.) Vill. and *Chenopodium album* L. [14]. In the latter case, the burial of seeds due to soil tillage would be ineffective as a control method.

The knowledge about the germination of *B*. *subalternans* seeds submitted to the different environmental conditions is a meaningful way to understand how this weed species establish in agricultural fields. Thereby, our objective was to evaluate the germination of *B*. *subalternans* seeds submitted to different environmental factors. We seek to answer which environmental conditions favor *B*. *subalternans* germination. Thereby, we will discuss how agricultural and cultural practices can help the control of this species.

## Materials and methods

### Seed collection and species identification

The seeds of *B*. *subalternans* (Black prick) were collected at the experimental farm in Rio Grande do Norte Agricultural Research Company (EMPARN), Parnamirim, RN (5°55'23.198''S, 35°12'16.859''O) during March 2017. The seeds were collected in an agricultural area with 25.000 m^2^, cleaned, and stored in a closed container at temperature (20°C) until used in the experiments. Crops grown on the site from August 2015 to July 2017 were maize, sorghum, and cotton. A random sampling of the plants was performed, selecting those with an average of 70% mature seeds (seeds showing black coloration and 15 mm in length). The number of plants sampled was 50 (approximately one plant per 500 m^2^). The collected seeds were manual cleaning. After this procedure, a subsample was performed containing 150 seeds.

The seeds were sowed in vessels and maintained in a greenhouse to monitor all development phases of the plants. The morphological characteristics of the plants were analyzed for the correct taxonomic classification [15]. After identification, the seeds were harvested, processed, packed, and stored in hermetically sealed containers and controlled conditions (20 ± 2°C and 50 ± 5% relative humidity) until the experiments were set up. Seed viability and dormancy were tested 15 days before the experiment execution. In this test was used four replicates of 50 seeds in plastic boxes (11-cm length, 11-cm width, and 3.5-cm height) containing two layers of blotting paper moistened with autoclaved distilled water. The water volume was equivalent to 2.5 times the dry weight of the paper. The germination was close to 90 ± 5%, and there was no dormancy in the seeds obtained from the sub-sampling.

### Germination test

The experiments were conducted in the laboratory during August to September 2017. The seeds were previously disinfected with 1% sodium hypochlorite for 1 minute and washed in distilled water. The germination test was performed in transparent plastic boxes (11-cm length, 11-cm width, and 3.5-cm height) containing two layers of blotting paper moistened with autoclaved distilled water. The water volume was equal to 2.5 times the dry weight of the paper [16]. Four replicates of 25 seeds were sown in the boxes. Subsequently, the boxes were placed in plastic bags of 0.05 mm thickness for moisture conservation, and kept in a Biochemical Oxygen Demand (B.O.D.) type germinator, adjusting the temperature and photoperiod according to preliminary tests (data not shown).

The seeds were classified as “germinated” when there was visible protrusion of the radicle at least 2‐mm long [17]. The first count for all experiments was performed on the third day after sowing. The light, temperature, and depth germination test were evaluated at 16 days after sowing (DAS) and the water and salt stress tests at 25 days DAS.

### Temperature effect

The experiment was conducted in controlled conditions. Five alternating temperature regimes (15/20°C, 20/25°C, 25/30°C, 30/35°C, and 35/40°C night/day) with the 12-hour light and dark photoperiod. These conditions simulated the mean, maximum, and minimum temperature variations that occur in the different Brazilian states (Table 1).

**Table 1:** Minimum, mean, and maximum temperatures in Brazilian regions during the years 2016 and 2017.

Brazilian regions	^a^ Temperature (ºC)					
	2016			2017		
	Minimum	Mean	Maximum	Minimum	Mean	Maximum
Northeast	30 ±2	32.5	36 ±2	30 ±2	32.5	35 ±2
North	30 ±2	32.5	35 ±3	31 ±2	32.5	37 ±2
Midwest	18 ±2	24	30 ±2	18 ±2	24	30 ±2
Southeast	14 ±4	20.5	27 ±2	12 ±3	19	26 ±2
South	8 ±2	16	24 ±2	8 ±2	16	24 ±4

^a^ Data were obtained from http://www.inmet.gov.br/portal/index.php?r=estacoes/mapaEstacoes.

The chosen temperature for the other tests (depth germination, water and salt stress) was 25/30°C due to higher values of germination and uniformity observed for this temperature interval.

### Light effect

To verify the light effect on seed germination of *B*. *subalternans*, light and dark regimes of 24/0, 12/12 and 0/24 hours were implemented under alternating temperature of 25/30°C night/day. The light intensity was 27 to 33.75 μmol m^-2^ s^-1^ provided by a lamp with white light (2000 lux). For the dark treatment, the plastic boxes were covered with two layers of aluminum paper to prevent that the light reaches the seeds. In this condition, the water additions and germination evaluations were performed in a dark environment under a green light.

The light regime selected to conduct the other tests was 12/12 hours (light/dark) due to the highest seed germination. Also, this regime simulates the conditions that generally occurs in tropical regions.

### Sowing depth

Seeds of *B*. *subalternans* were sown at depths of 0, 2, 4, 6, and 8 cm in a polyethylene vessel (25-cm length, 25-cm width, 9-cm height) and filled with inert material (sand washed and sterilized in oven at 200°C for 2 hours). The alternating temperature and photoperiod used during this test was 25/30°C night/day and 12 hours, respectively.

### Water stress

To verify the effect of water stress on germination of *B*. *subalternans*, aqueous solutions of polyethylene glycol 6000 (PEG) [HO(C₂H₄O)nH] were prepared with osmotic potentials of 0.0, -0.1, -0.2, -0.3, and -0.4 MPa (concentrations of 0.0, 78.5, 119.6, 151.4, 178.3 mM, respectively), according to the recommendations of Munns and James [16] under alternating temperature of 25/30°C night/day with 12 hours of photoperiod.

### Salt stress

The effect of saline stress on germination of *B*. *subalternans* was evaluated in aqueous solutions, humidifying germitest papers with distilled water (control) or sodium chloride (NaCl) solutions. The water potential was 20.18, 40.36, 60.54, 80.72, and 100.09 mM, related to concentrations of (-0.1, -0.2, -0.3, 0.4 and 0.5 MPa). Salt stress tests were conducted at alternating temperature 25/30°C night/day with 12 hours of photoperiod.

### Statistical analyses

The experiments were repeated twice and organized into the completely randomized design, with four replications. The second series of experiments was carried out fifteen days after the end of the first. The transformation of the data into a square or logarithmic root did not improve the homogeneity of the variance; ANOVA was performed in germination percentage values not transformed (Statistix statistical software v. 8.1). No statistical significance (p≤0.05) was found for the factor Experiments (Experiment 1 and 2); therefore, the data of experiment 1 and 2 were grouped and used for subsequent analyzes. The means of the treatments of each experiment were shown in scatter plots with the bars indicating the least significant difference (LSD, p≤0.05).

## Results and discussion

### Effect of temperature on germination

**Fig 1:**
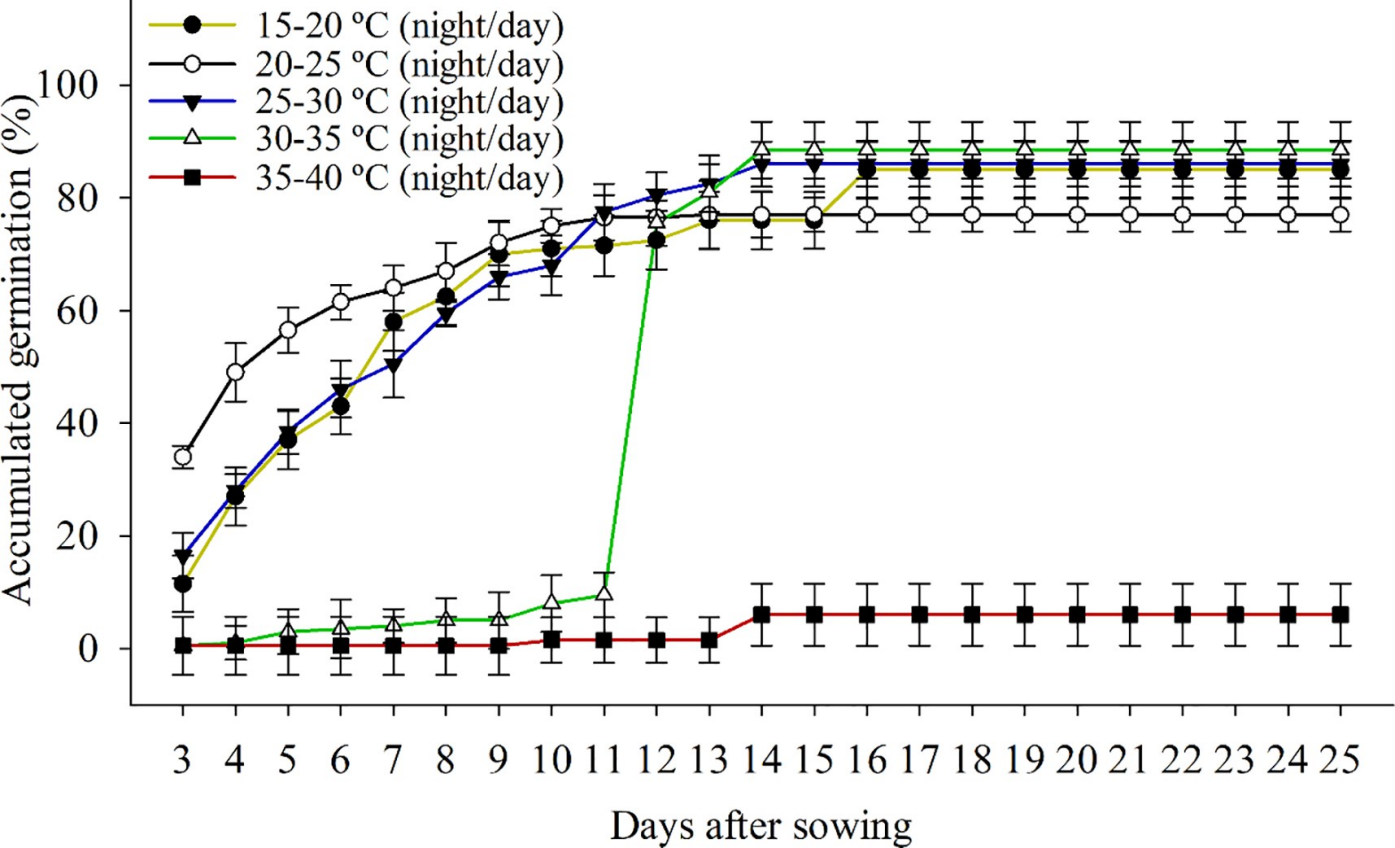
Seed germination of *Bidens subalternans* DC. at different temperatures (vertical bars represent the least significant difference, LSD, p≤0.05).

In low-temperature conditions, the *B*. *subalternans* showed a constant increase in the germination until 14th day (Fig 1). The highest temperature reduced the germination of *B*. *subalternans*, with values extremally low (Fig 1). This behavior indicates that this low-temperature range favors the germination of *B*. *subalternans*. Probably, higher temperature stimulates biosynthesis of abscisic acid (ABA) and antagonizes synthesis of gibberellins (GA) inhibiting seed germination of *B*. *subalternans* [18].

The temperatures of 15/20, 20/25, 25/30, and 30/35°C (night/day) provided the highest germination of the *B*. *subalternans* seeds, with values of 85%, 77%, 86%, 88%, respectively (Fig 1). The temperatures of 35/40°C (night/day) caused the lowest seed germination (11%) (Fig 1). The seeds of *B*. *subalternans* presented a high germination rate at a wide temperature range, varying from 15 to 35°C during the day (Fig 1). This fact explicit the dispersion and infestation pattern of this species during all year in different Brazilian regions [19, 20, 21, 22].

Despite the *B*. *subalternans* show flexibility to germinate at different temperatures, the high temperature impaired the germination process. Temperatures of 30/35 and 40/35°C (night/day) reduced the germination process from the eleventh day. Studies have shown that temperatures above 35°C reduced the germination of several weed species [23, 24, 25, 26], similar to the seeds of *B*. *subalternans*. Ramirez et al. [27] evaluating the *Bidens alba*, species belonging to the same genus *Bidens*, reported that the optimum temperature for germination was 20/25°C, and those higher than 30°C inhibited the germination in 60%. Differently, *B*. *subalternans* can germinate at a higher temperature (30/35°C), with optimum values observed in 25/30°C. Although they belong to the same genus, these species may have different physiological and biochemical mechanisms that give better adaptation to the different temperatures range. This fact can be evidenced by observing the regions where the seeds were collected. The *B*. *alba* seeds were collected in a site with minimum and maximum temperatures of 12 and 23°C. In contrast, the *B*. *sulbalternans* seeds were collected in Brazilian northeastern, a region with high temperatures (25 to 35°C).

Other species belonging to family Asteraceae were subjected to temperatures higher than 35°C and the germination was inhibited due to thermo-inhibition mechanism via a set of genes [17, 18, 28, 29]. Previous studies showed that the 9-cis-epoxycarotenoid dioxygenase gene was able to stimulate ABA synthesis in seeds at temperatures above 30°C [28, 29]. The elevation in ABA levels promotes inhibiting gibberellin activity, an essential hormone that activates the germinative process [30]. Also, high temperatures can inhibit the ethylene synthesis, another hormone responsible for inducing the seed germination [18]. Thus, similar mechanisms may be present in *B*. *subalternans* seeds, causing the inhibition of germination under high temperatures.

In the northeast Brazil, soybeans and corn cultivated between October and November, when the temperature reaches 25 to 35°C, may increase the infestation this species due to ideal conditions for germination of *B*. *subalternans*. The synchronization of these two factors increases the importance of this weed because the germination of *B*. *sulbalternans* will occur during the stages in which the cultures show higher sensitivity to interference [31].

### Effect of light and burial depth on germination

**Fig 2:**
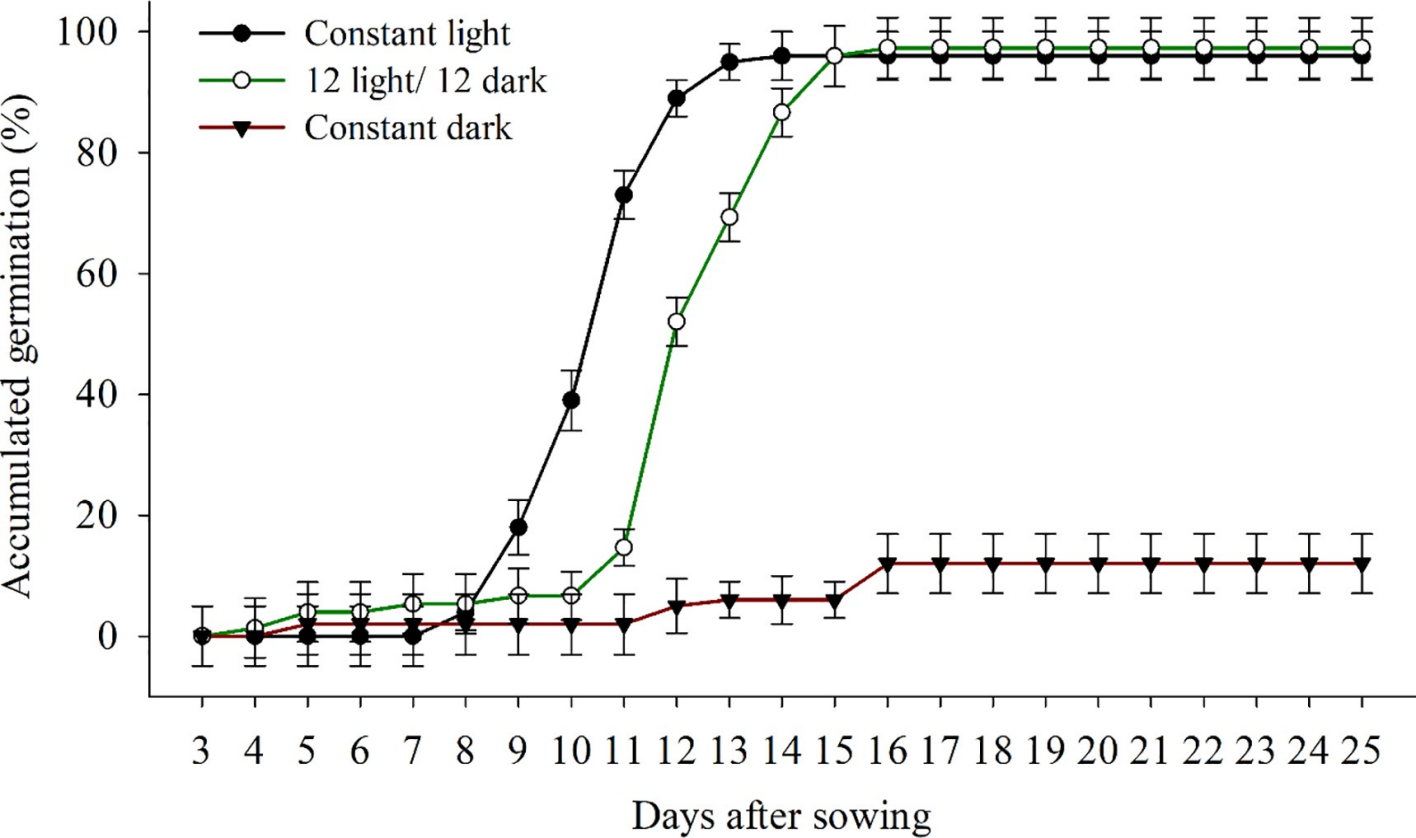
Seed germination of *Bidens subalternans* DC. in different photoperiods (vertical bars represent the least significant difference, LSD, p≤0.05).

The seeds exposed to 12 h light/dark conditions showed maximum germination of 96% on the 16th day (Fig 2). However, in constant light, the germination of *B*. *subalternans* reached 96% on the 13th day (Fig 2). The lowest germination percentage was verified for treatments under constant dark, with an inhibition close to 83% (Fig 2). The slower germination speed of *B*. *subalternans* in 12 h light/dark treatments compared to constant light indicates that the time of light exposure also affects the germination percentage.

The germination for treatments exposed to 12 hours light/dark probably is due to the active-state of "phytochrome-Pfr" in *B*. *subalternans* seeds. However, the "phytochrome-Pfr" can convert for their inactive-state (phytochrome-Pf) in dark conditions, impairing the germination. Thus, higher exposure to solar irradiation is necessary to favor the relationship between phytochrome-Pfr/phytochrome-Pr that stimulate germinative processes [32]. This behavior has already been verified in several weeds species, is known as species with low fluence response (LFR) [33].

**Fig 3:**
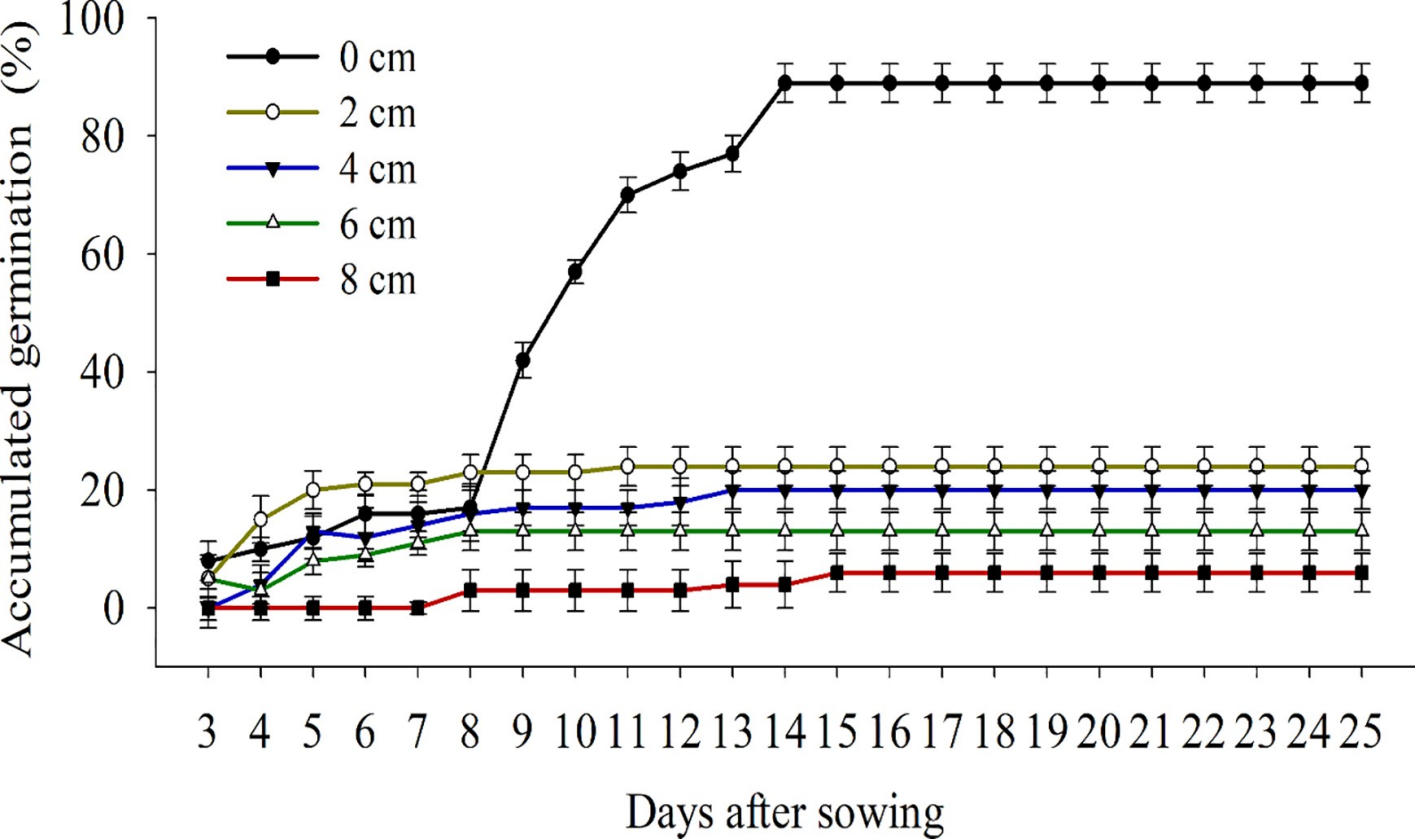
Emergency of *Bidens subalternans* DC. exposed to different seeding depths (vertical bars represent the least significant difference, LSD, p≤0.05).

The role of light as inducer on the germination of *B*. *subalternans* seeds was confirmed during the burial depth test. The seeds of this weed showed higher germination when sown on the soil surface compared to 2, 4, 6, and 8 cm depths (Fig 3). The seed germination percentage at 2 cm burial depth was only 22%, and values close to zero for those at 8 cm deep (Fig 3).

These results indicate that no-tillage or minimum planting can favor the establishment of *B*. *subalternans* because these systems maintained a more significant number of weed seeds on the soil surface [34]. On the other hand, practices such as plowing and harrowing (or any other one that promotes the seed burial) acts as a control method for this species. Studies have already shown that soil tillage can reduce the incidence of various weeds [34, 35, 36], and it also can be viable for *B*. *subalternans*. However, these practices have limited efficiency because frequent soil tillage may bring the buried seeds back to the soil surface.

### Effect of water stress on germination

**Fig 4:**
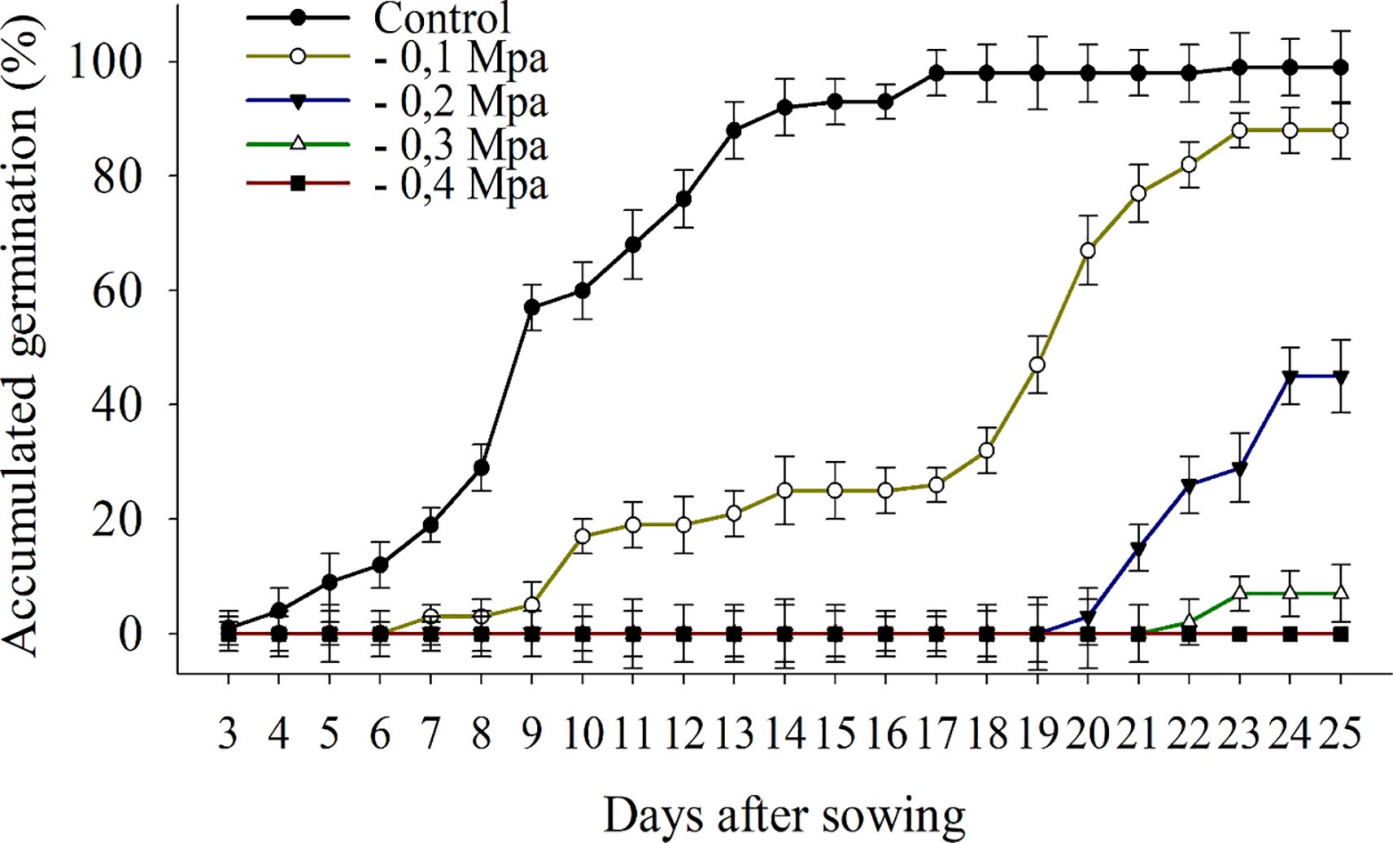
Germination of *Bidens subalternans* DC. exposed to different water potentials (vertical bars represent the least significant difference, LSD, p≤0.05).

The seeds exposed to water stress equal to -0.4 MPa did not germinate (Fig 4). The maximum germination of *B*. *subalternans* (99%) was observed in the control treatment at 17 DAS (Fig 4). However, water restrictions of -0.1 and -0.2 MPa reduced the germination in 12% and 45%, respectively, compared to the control, at 25 DAS (Fig 4). The water potential of -0.3 MPa decreased seed germination by 95% compared to the control (Fig 4).

The seeds of *B*. *subalternans* demonstrated difficulties to absorb water under conditions of -0.2 MPa since a low germination percentage was verified for this water potential. *B*. *subalternans* seeds did not germinate under -0.4 MPa, indicating the high sensitivity of this species to the water deficit compared to other weeds. Species, such as *Eupatorium adenophorum* L. [37], *Bromus japonicus* L. [25], and *Sophora alopecuroides* L. [13], showed higher tolerance to water deficit and only had germination inhibited when exposed to water potentials below -0.7 MPa, -1.3 MPa and -1.5 MPa, respectively.

The highest infestation of *B*. *subalternans* in agricultural fields is observed during the summer in central-west, south-east, and south Brazilian regions, season with high rainfall incidence [38]. Although higher temperatures disfavor the germination of *B*. *subalternans* seeds, the water restriction is the most limiting factor for the germination of this species. Therefore, regions and climate conditions with a higher occurrence of rainfall favor the establishment of *B*. *subalternans*.

### Effect of salt stress on germination

**Fig 5:**
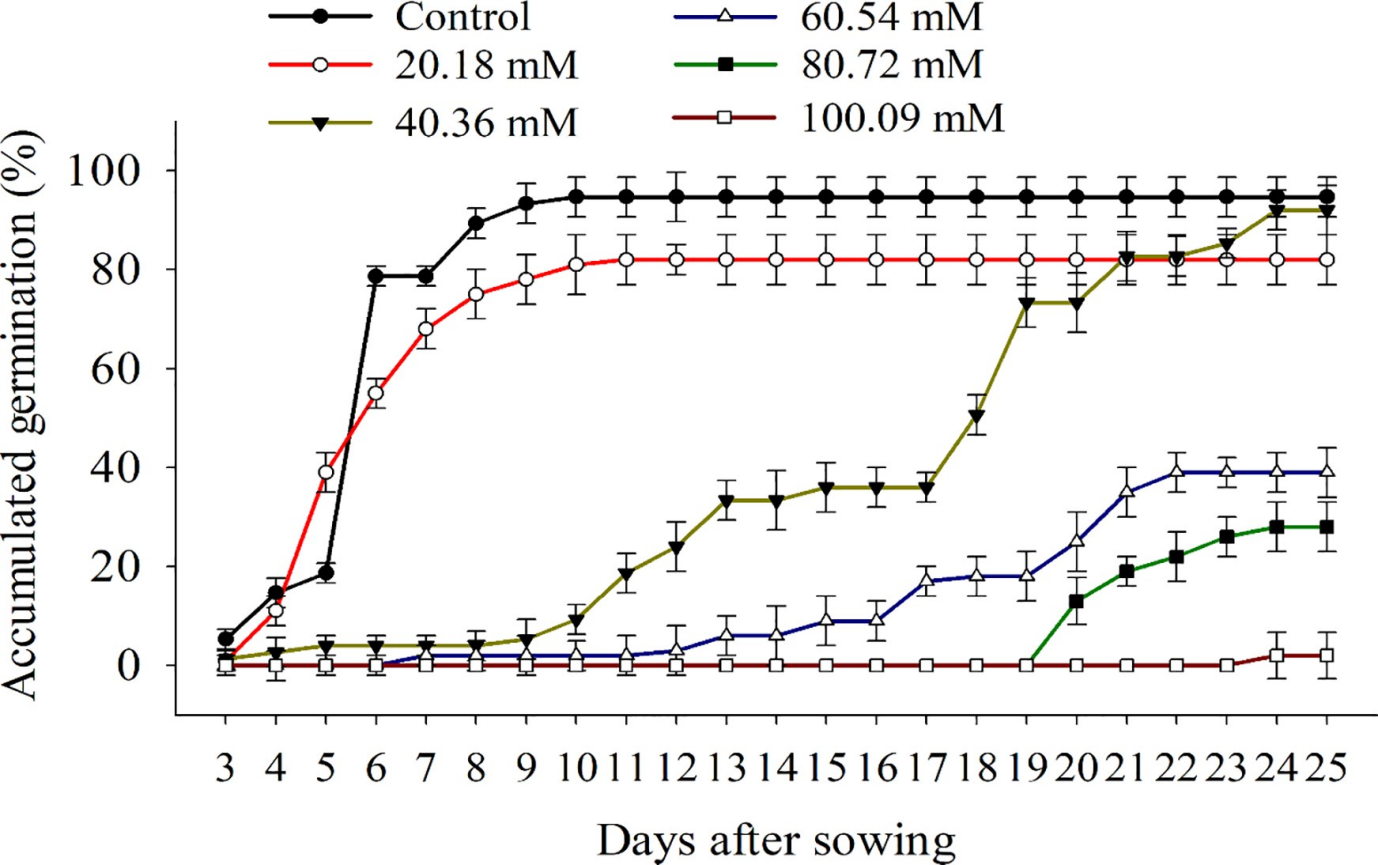
Germination of *Bidens subalternans* DC. submitted to salt stress (vertical bars represent the least significant difference, LSD, p≤0.05).

The increase in salt concentration reduced the seed germination of *B*. *subalternans* (Fig 5). The seeds did not germinate in salt stress equal to 100.09 mM (Fig 5). The control treatment presented germination higher than 94% at 10 DAS. In osmotic potentials of 20.18 mM and 40.36 mM, the seed germination was 86% and 92%, respectively. Although the higher germination, the water potential of 40.36 mM reduced the speed of germination (Fig 5). The osmotic potential of 60.54 mM inhibited the germination in 60%, and plots treated with 100.09 mM showed complete inhibition.

The reduction and delay of the germinative process in high saline concentrations may have occurred due to the decrease of the free water energy [39]. Reduction of water potential due to NaCl addition impairs water absorption by seed, and those species more sensitive to water stress, as *B*. *subalternans*, are generally sensitive to the saline environments [40]. Other weed species, such as *Chamaesyce maculate* (L.) Small [41] and *Cucumis melo* L. [42], showed a higher capacity to tolerate saline stress compared to the *B*. *subalternans*, presenting germination equivalent to 80% even when subjected to 80.72 mM and 141.26 mM NaCl, respectively.

The seeds of *B*. *subalternans* were more sensitive to water deficit (Fig 5) than to saline stress (Fig 5). The seed germination in the presence of NaCl was higher than PEG, considering solutions with similar osmotic potentials. The ions intake by seeds can decrease the internal osmotic potential and allow the absorption of water by the *B*. *subalternans* seed, initiating the germination [43]. Even though the sensitivity of *B*. *subalternans* to conditions of salt and water stress have been reported in this work, molecular and biochemical studies must be conducted to fully identify the mechanisms involved in the susceptibility of this species. Once the genes and mechanisms have been identified, it is possible to trace studies that demonstrate the genetic differences existing in biotypes of *B*. *subalternans*.

## Conclusion

This study demonstrated that abiotic factors exerted a significant influence on the germination of *B*. *subalternans* seeds and contributed to understanding the ecology of this species. Germination capacity in a wide range of temperatures favors the occurrence of this weed throughout the Brazilian territory. However, the sensitivity to water and salt stress explain the higher incidence of *B*. *subalternans* in south-central Brazilian states where is observed high rainfall frequency. The test about the effect of luminosity and sowing depth showed that the burial could reduce the infestations of *B*. *subalternans*. At the same time, the no-tillage increases the germination of *B*. *subalternans* because the seeds would be exposed to the soil surface, a condition that favors the germination.
